# mTORC1 beyond anabolic metabolism: Regulation of cell death

**DOI:** 10.1083/jcb.202208103

**Published:** 2022-10-25

**Authors:** Jiajun Zhu, Hua Wang, Xuejun Jiang

**Affiliations:** 1 Department of Basic Medical Sciences, School of Medicine, Tsinghua University, Beijing, China; 2 Tsinghua-Peking Center for Life Sciences, Beijing, China; 3 Cell Biology Program, Memorial Sloan Kettering Cancer Center, New York, NY

## Abstract

The mechanistic target of rapamycin complex 1 (mTORC1), a multi-subunit protein kinase complex, interrogates growth factor signaling with cellular nutrient and energy status to control metabolic homeostasis. Activation of mTORC1 promotes biosynthesis of macromolecules, including proteins, lipids, and nucleic acids, and simultaneously suppresses catabolic processes such as lysosomal degradation of self-constituents and extracellular components. Metabolic regulation has emerged as a critical determinant of various cellular death programs, including apoptosis, pyroptosis, and ferroptosis. In this article, we review the expanding knowledge on how mTORC1 coordinates metabolic pathways to impinge on cell death regulation. We focus on the current understanding on how nutrient status and cellular signaling pathways connect mTORC1 activity with ferroptosis, an iron-dependent cell death program that has been implicated in a plethora of human diseases. In-depth understanding of the principles governing the interaction between mTORC1 and cell death pathways can ultimately guide the development of novel therapies for the treatment of relevant pathological conditions.

## Teaser

Zhu et al. review the current knowledge on how mTORC1 activity is connected to cell death regulation through metabolic and signaling pathways.

## Introduction

Mechanistic or mammalian target of rapamycin (mTOR) is a member of the phosphoinositide 3-kinase (PI3K)-related serine/threonine protein kinase family (Battaglioni et al., 2022; Liu and Sabatini, 2020). In mammalian cells, mTOR acts as the catalytic subunit in two distinct complexes termed mTORC1 and mTORC2, of which mTORC1 is characterized as a central coordinator of cellular metabolic homeostasis. In the presence of growth-promoting signals and sufficient intracellular nutrient supply, mTORC1 is activated to favor biosynthesis of proteins, lipids, and nucleic acids, while suppressing catabolism of these macromolecules. By contrast, nutrient limitation or a lack of growth factor signaling dampens mTORC1-mediated anabolic processes but leads to enhanced lysosomal degradation of macromolecules of both intracellular and extracellular sources.

Cellular metabolic homeostasis underlies all essential biological activities, in part, by providing the substrates and energy required to maintain intracellular biochemical reactions (Judge and Dodd, 2020; Zhu and Thompson, 2019). A mismatch of metabolic supply with cellular demand, due to environmental insult or pathological abnormality, can lead to various stress responses, including the execution of cell death programs. In the past decade, ferroptosis has emerged as a distinct type of regulated cell death intimately connected to cellular metabolism (Jiang et al., 2021a; Stockwell, 2022). Ferroptosis is iron-dependent and is often preceded by a dysregulated cellular redox state that leads to an increase in lipid reactive oxygen species (ROS). Remarkably, multiple specific oncogenic mutations alter cellular metabolism and hence the sensitivity of cancer cells to ferroptosis, making ferroptosis induction a promising cancer therapeutic approach, either alone or in combination with other regimens. On the other hand, ferroptosis appears to be an important pathological contributor to various degenerative diseases, suggesting the therapeutic potential of ferroptosis inhibition. In many of these cases, mTORC1 has been demonstrated as an important node in determining ferroptotic activity through its regulation of the intracellular metabolic network.

In this review, we will analyze the expanding evidence that connects mTORC1 activity with ferroptosis through specific metabolites and downstream signaling, transcriptional, and epigenetic events. We will also briefly summarize the role of mTORC1 signaling in other forms of regulated cell death, including apoptosis and pyroptosis. Furthermore, the potential of exploiting the connection between mTORC1 and ferroptosis for disease treatment will be discussed.

### Metabolites that coordinate mTORC1 activity with ferroptosis regulation

Ferroptosis was defined a decade ago as an iron-dependent, non-apoptotic form of cell death (Dixon et al., 2012). Ferroptotic death is driven by iron-dependent peroxidation of phospholipids (PL) containing polyunsaturated fatty acid (PUFA) tails (Yang et al., 2016; Yang and Stockwell, 2016). As such, ferroptosis can be potently induced by depletion of intracellular cysteine required for glutathione (GSH) biosynthesis, or by perturbation of the glutathione peroxidase 4 (GPX4) enzymatic activity that utilizes GSH to catalyze the reduction of PUFA-PL peroxides (Dixon et al., 2012; Gao et al., 2015; Yang et al., 2014). Extending from these observations, a variety of intracellular metabolites and metabolic processes have been directly implicated in the progression of ferroptosis, many of which are components of mTORC1 regulated pathways.

#### Amino acid

**Figure 1.**
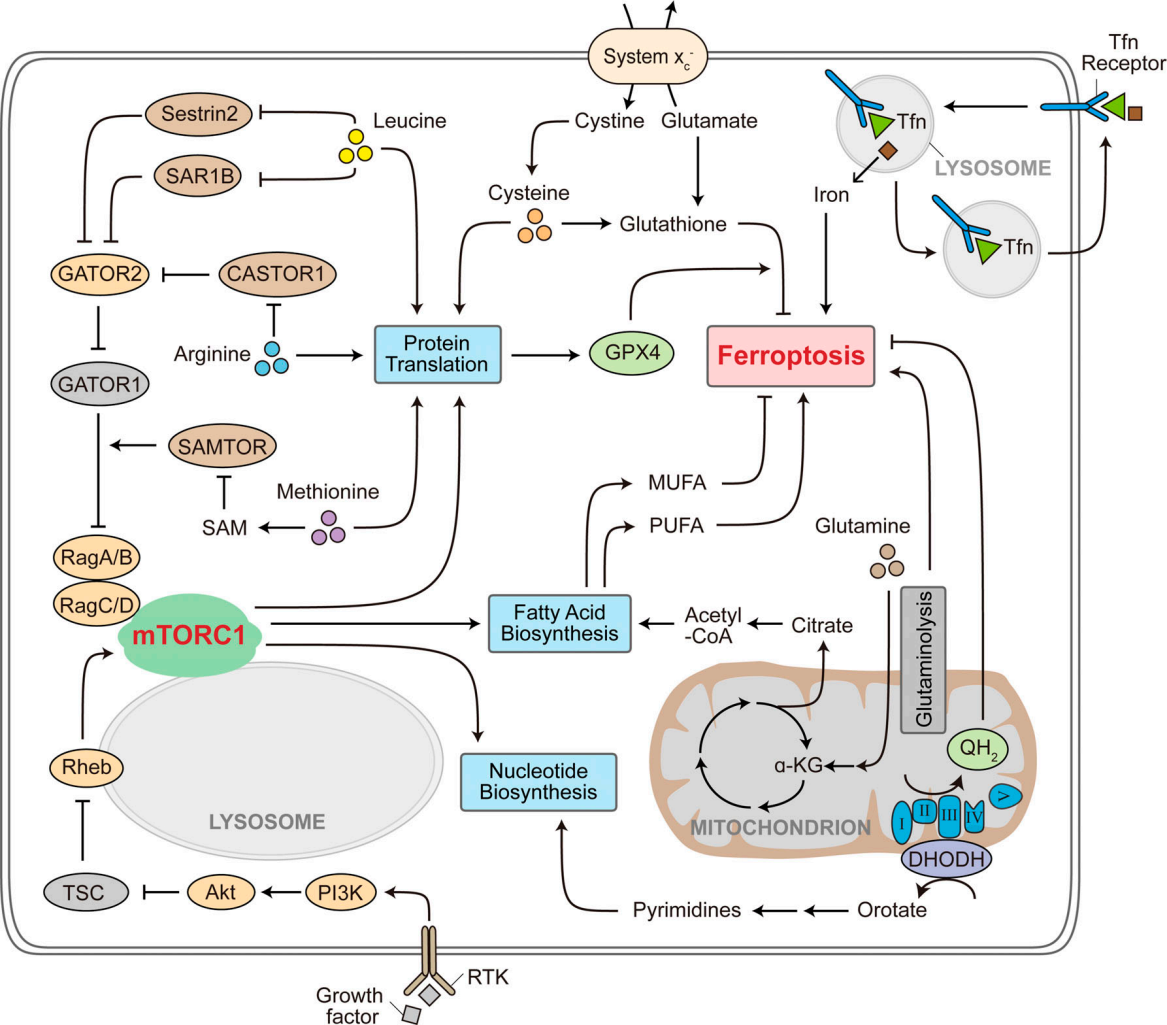
**Metabolites that coordinate mTORC1 activity with ferroptosis regulation.** mTORC1 interrogates growth factor signaling and cellular nutrient status to regulate anabolic metabolism including protein translation, fatty acid biosynthesis, and nucleotide biosynthesis. mTORC1 senses the availability of leucine, arginine, and SAM (indicative of methionine abundance) through the Sestrin2 and SAR1B, CASTOR1, and SAMTOR sensor proteins, respectively. Intracellular cysteine contributes to ferroptosis resistance by supporting both glutathione biosynthesis and GPX4 protein synthesis. By contrast, intracellular glutamine utilization by glutaminolysis sensitizes ferroptosis. Production of MUFA leads to ferroptosis resistance, whereas PUFA generation promotes ferroptosis. Pyrimidine synthesis is coupled to the cellular energy status through DHODH that functions at the electron transport chain (ETC). DHODH mediates orotate production while depositing electrons to the ETC for generation of ubiquinol, which acts as a suppressor of ferroptosis. Transferrin receptor-mediated iron uptake and subsequent iron release from the lysosome promote ferroptosis execution. RTK, receptor tyrosine kinase. PI3K, phosphoinositide 3-kinase. TSC, tuberous sclerosis complex. MUFA, monounsaturated fatty acid. PUFA, polyunsaturated fatty acid. DHODH, dihydroorotate dehydrogenase. QH_2_, ubiquinol. Tfn, transferrin.

Amino acids are building blocks of protein synthesis and substrates involved in other biosynthetic and bioenergetic activities. mTORC1 interrogates the information of intracellular amino acid availability through several dedicated sensor proteins that function as negative regulators of mTORC1. For instance, Sestrin2 and SAR1B, CASTOR1, and SAMTOR were identified as sensor proteins of leucine, arginine, and S-adenosylmethionine (SAM, indicative of methionine level), respectively (Fig. 1). The presence of leucine, arginine, or methionine alleviates the inhibitory effect of their cognate sensor proteins mediated through the GATOR complexes, thereby permitting activation of the Rag heterodimer and subsequent recruitment of mTORC1 to its site of action—lysosomes (Fig. 1). By contrast, deprivation of any of these amino acids results in an inactive configuration of the Rag heterodimer and thereby dissociation of mTORC1 from the lysosomes (Chantranupong et al., 2016; Chen et al., 2021c; Gu et al., 2017; Saxton et al., 2016a; Saxton et al., 2016b; Wolfson et al., 2016). In addition, the deficit of amino acids in general, beyond that of leucine, arginine, and methionine, can lead to the integrated stress response (ISR) that dampens mTORC1 activity (Ye et al., 2015).

Cysteine/cystine is an essential nutrient for survival and growth of cultured cells (Eagle, 1955). Cysteine deprivation causes rapid cell death that can be prevented with antioxidant supplementation (Bannai et al., 1977). Reminiscent of these observations, screening of a library of small molecules and subsequent mechanistic investigation revealed the chemical compound erastin as a potent inducer of ferroptosis by inhibiting the system x_c_^−^ plasma membrane antiporter, which mediates cystine uptake in exchange for intracellular glutamate (Dixon et al., 2012; Fig. 1). Consistent with the role of cysteine in supporting cell survival and growth, availability of cysteine was reported to be essential to maintain mTORC1 function (Daher et al., 2019; Yu and Long, 2016). Paradoxically, during prolonged starvation in *Drosophila* larva, lysosome-derived cysteine limits mTORC1 reactivation by retaining nutrient utilization in the mitochondrial tri-carboxylic acid (TCA) cycle (Jouandin et al., 2022). Whether this repressive role of cysteine in mTORC1 regulation requires additional metabolic regulations that are specific to *Drosophila* larval fat body is an intriguing topic for future investigation. Interestingly, system x_c_^−^-mediated cystine uptake promotes GPX4 protein translation in an mTORC1-dependent manner, whereas inhibition of mTORC1 reduces GPX4 production and synergized with ferroptosis induction in cancer cells (Zhang et al., 2021). Further, mTORC1 promotes activating transcription factor 4 (ATF4) activity and upregulates the expression of SLC7A11, the catalytic subunit of system x_c_^−^ (Torrence et al., 2021). All these studies demonstrate a complex interplay of mTORC1 and cysteine availability in the regulation of ferroptosis sensitivity.

In addition to being a building block for protein synthesis, glutamine plays a multifaceted role in cellular metabolism by supporting nucleotide biosynthesis, non-essential amino acid production, and the TCA cycle anaplerosis (Pavlova and Thompson, 2016). This is likely consistent with the existence of multiple mechanisms by which glutamine can signal to mTORC1 activity. In addition to the Rag heterodimeric GTPase, glutamine availability is sensed by mTORC1 through the adenosine diphosphate ribosylation factor-1 (Arf1) GTPase (Bernfeld et al., 2018; Jewell et al., 2015; Meng et al., 2020), and through the SLC7A5/SLC3A2 heterodimeric antiporter that imports essential amino acids at the expense of glutamine efflux (Nicklin et al., 2009). Glutamine also activates mTORC1 through glutaminolysis, in a α-ketoglutarate (α-KG)-dependent manner (Duran et al., 2012). Intriguingly, while cysteine deprivation promotes ferroptosis, absence of glutamine, or disruption of the glutaminolysis pathway leads to ferroptosis resistance (Gao et al., 2015; Fig. 1). Consistent with this, microRNA targeting the glutamine importer SLC1A5 antagonizes ferroptosis in melanoma cells (Luo et al., 2018). The ferroptosis-promoting effect of glutaminolysis is, at least in part, due to an increase in the activity of mitochondrial TCA cycle and electron transport chain (Gao et al., 2019). Whether mTORC1 plays a direct role in mediating the function of glutamine in ferroptosis will be an interesting question to explore.

Similar to the effect of glutamine deprivation, ferroptosis induced by erastin or cysteine withdrawal can be suppressed when cells are cultured in medium lacking arginine, lysine, valine, or methionine, but not when deprived of other amino acids such as glycine, phenylalanine, tryptophan, or serine (Conlon et al., 2021). Although precise mechanisms underlying the differential effect of these individual amino acids on ferroptosis are not clear, it was suggested to be independent of mTORC1 inhibition or ATF4 induction, but likely mirrors the distinct role of individual amino acids on cell proliferation (Conlon et al., 2021).

#### Lipid and cholesterol

mTORC1 promotes lipid and cholesterol production by activating transcriptional programs mediated by the sterol regulatory element-binding protein 1 (SREBP1) and SREBP2. This is accomplished partly through phosphorylation of S6K1 (encoded by *RPS6KB1*), lipin 1 (encoded by *LPIN1*), and CREB-regulated transcription coactivator 2 (CRTC2; Duvel et al., 2010; Han et al., 2015; Owen et al., 2012; Peterson et al., 2011; Porstmann et al., 2008). mTORC1 also increases mRNA stability of lipogenic enzymes downstream of SREBPs through SRPK2-mediated pre-mRNA splicing, and increases protein stability of the cholesterogenic enzyme HMGCR through phosphorylation of its deubiquitinase USP20 (Lee et al., 2017; Lu et al., 2020). Expansion of the cellular lipogenic and cholesterogenic capacity is associated with an active mTORC1 state instructed by growth-promoting signals and sufficient nutrients.

Lipid peroxidation underlies ferroptosis progression. PL containing PUFA tail(s) are the major substrates that initiate and propagate lipid peroxidation, through both the nonenzymatic Fenton reaction and the lipoxygenases-catalyzed lipid radical formation (Liang et al., 2022). As a result, acyl-CoA synthetase long-chain family member 4 (ACSL4) that ligates coenzyme A (CoA) to PUFA for subsequent PUFA-PL formation is crucial for ferroptosis execution (Doll et al., 2017; Kagan et al., 2017). In contrast to a ferroptosis-promoting role of PUFAs, monounsaturated fatty acids (MUFAs) inhibit ferroptosis in an ACSL3-dependent manner (Magtanong et al., 2019; Fig. 1), although further mechanistic details underlying this effect of MUFAs remain to be defined.

mTORC1 activation, such as in the context of oncogenic phosphoinositide 3-kinase (PI3K) mutation or PTEN deletion, can lead to ferroptosis resistance in cancer cells by activating SREBP1, which in turn upregulates stearoyl-CoA desaturase 1 (SCD1) to catalyze MUFA production (Yi et al., 2020). It was further demonstrated that the combination of mTORC1 inhibition with ferroptosis induction resulted in substantial tumor regression in mouse models of PI3K-mutated or PTEN-defective cancers (Yi et al., 2020). As ferroptosis-inhibiting role of MUFAs was consistently observed in other contexts such as ovarian cancer and metastatic melanoma (Tesfay et al., 2019; Ubellacker et al., 2020), targeting MUFA production might represent a promising therapeutic strategy to sensitize cancer cells to ferroptosis induction.

Similar to PUFA, cholesterol is capable of generating and propagating peroxidized products in cellular membrane, hence contributing to ferroptosis (Girotti and Korytowski, 2019; Thomas et al., 1990a; Thomas et al., 1990b). In addition, the mevalonate pathway that mediates de novo cholesterol biosynthesis is intrinsically connected with ferroptosis regulation. The mevalonate pathway intermediate, isopentenyl pyrophosphate (IPP), regulates selenocysteine tRNA maturation by adenosine isopentenylation, which is essential for the biosynthesis of selenoproteins, including GPX4 (Warner et al., 2000). Moreover, accumulation of squalene due to suppressed squalene monooxygenase expression in a subset of anaplastic large cell lymphomas renders these tumors cholesterol auxotrophic but also resistant to ferroptosis (Garcia-Bermudez et al., 2019). Chronic exposure to a high level of cholesterol increases GPX4 expression in breast cancer cells, resulting in ferroptosis resistance and elevated tumorigenicity (Liu et al., 2021). On the other hand, cholesterol in the tumor microenvironment promotes CD36-mediated fatty acid uptake in CD8^+^ T cells, leading to increased ferroptosis in T cells and hence dampened tumor suppression (Ma et al., 2021). Although mTORC1 can impact cholesterol synthesis through regulating SREBPs, whether it plays a direct role in connecting cholesterol biosynthesis to ferroptosis remains to be determined.

#### Nucleotide

In addition to promoting protein and lipid production, activation of mTORC1 stimulates de novo nucleotide biosynthesis. mTORC1 phosphorylates and activates S6K1, which in turn phosphorylates the multi-domain enzyme CAD (carbamoyl-phosphate synthetase 2, aspartate transcarbamylase and dihydroorotase), leading to its oligomerization and consequent increase of pyrimidine synthesis (Ben-Sahra et al., 2013; Robitaille et al., 2013). In addition, mTORC1 promotes mitochondrial folate cycle activity, which in turn contributes one-carbon units to enhance purine synthesis (Ben-Sahra et al., 2016). Conversely, availability of purine regulates mTORC1 activity through both the tuberous sclerosis complex (TSC) and the Rheb GTPase activity (Emmanuel et al., 2017; Hoxhaj et al., 2017). Elevated nucleotide biosynthesis by mTORC1 activation is required during cancer initiation and progression, and has become a targetable tumor vulnerability (Buj et al., 2019; Valvezan et al., 2020; Valvezan et al., 2017).

Dihydroorotate dehydrogenase (DHODH) is localized at inner mitochondrial membrane and connects pyrimidine synthesis with mitochondrial metabolism. In the pyrimidine synthesis pathway, DHODH oxidizes dihydroorotate to produce orotate while transferring the electrons to ubiquinone (producing ubiquinol) as part of the mitochondrial electron transport chain (Fig. 1). Therefore, orotate production for pyrimidine synthesis is coupled to mitochondrial oxidative phosphorylation and the cellular energy status. Because ubiquinol is a radical-trapping antioxidant in mitochondria, its production mediated by DHODH is a major ferroptosis defense mechanism (Mao et al., 2021; Fig. 1). DHODH inhibition was further found to synergize with system x_c_^−^ suppression in ferroptosis induction (Mao et al., 2021). By contrast, conversion of ribonucleotides to deoxyribonucleotides by ribonucleotide reductase (RNR) consumes GSH. As a result, inhibition of RNR led to ferroptosis resistance by conserving GSH for antioxidant defense (Tarangelo et al., 2022).

#### ROS

Active anabolic metabolism is usually associated with ROS generation. Consistently, mTORC1 activation often leads to increased intracellular ROS levels. For example, activation of platelet derived growth factor receptor (PDGFR) promotes ROS generation in an mTORC1-dependent manner (Lei and Kazlauskas, 2014). Similarly, in chronic myelogenous leukemia carrying BCR-ABL fusion, PI3K-mTORC1 signaling downstream of BCR-ABL receptor tyrosine kinase (RTK) activity increases ROS through elevated glycolysis and mitochondrial respiration (Kim et al., 2005). In the context of hematopoietic stem cells (HSCs), TSC1 deletion was found to unleash mTORC1 activity, which in turn promoted ROS generation and contributed to the HSC transition from quiescence into a rapid cycling state (Chen et al., 2008).

Excessive ROS, especially when acting on PL, potentiates ferroptosis. However, a modest increase in ROS level can stimulate cell proliferation, partly due to a role of hydrogen peroxide in suppressing protein tyrosine phosphatases such as PTEN by oxidizing their catalytic cysteine residues (Diebold and Chandel, 2016; Tonks, 2005). In agreement, an increase in ROS level was shown to activate mTORC1 in a variety of biological settings, including cell proliferation, macrophage polarization, and inflammatory response (Formentini et al., 2017; Noh et al., 2019; Saxton et al., 2016b; Yalcin et al., 2010). Maintaining mTORC1 activation under ROS stimulation requires specific antioxidant mechanisms, such as elevated superoxide dismutase 1 (SOD1) and SOD2 expression, or sustained GSH biosynthesis (Gutierrez-Uzquiza et al., 2012; Mak et al., 2017). Therefore, mTORC1 activity and ROS level regulate each other, and that an imbalance of this mutual regulation may influence the execution of cell death.

#### Polyamine

Additional metabolites such as polyamines have emerged as connecting mTORC1 activity with ferroptosis regulation. In cancer cells harboring oncogenic Ras mutation, mTORC1 activity promotes ornithine decarboxylase (ODC) mRNA stability, thereby increasing the production of putrescine (Origanti et al., 2012). During polyamine biosynthesis, decarboxylated S-adenosylmethioinine (dcSAM) is further required to convert putrescine into spermidine and spermine. In prostate cancer, the frequent loss of PTEN and activation of mTORC1 leads to increased protein stability of S-adenosylmethionine decarboxylase 1 (AMD1), which catalyzes the production of dcSAM and facilitates polyamine biosynthesis (Zabala-Letona et al., 2017). Deregulated polyamine homeostasis has been suggested to contribute to ferroptosis. Spermidine/spermine N^1^-acetyltransferase 1 (SAT1) was identified as a transcriptional target of the p53 tumor suppressor. Upregulated SAT1 promotes catabolism of polyamines, which generates peroxide as a byproduct and therefore contributes to p53-mediated ferroptosis induction (Ou et al., 2016).

#### Iron-sulfur cluster (ISC)

ISC biogenesis is an essential component of various biological processes as ISC-containing proteins participate in mitochondrial electron transport, DNA damage repair, and protein translation (Rouault, 2015). Unsurprisingly, mTORC1 phosphorylates the scaffold protein ISCU to facilitate ISC biogenesis (La et al., 2013). ISC biogenesis is coupled to the availability of intracellular iron and is a critical determinant of ferroptosis. ISC biosynthesis requires the coordinated action of multiple regulatory proteins, including the NFS1 cysteine desulfurase, as well as the Frataxin (FXN) protein that functions as an allosteric activator of the NFS1 complex (Bridwell-Rabb et al., 2014; Land and Rouault, 1998; Parent et al., 2015; Rouault, 2015). Disruption of ISC biosynthesis by genetic inhibition of FXN or NFS1 led to iron-starvation response that promotes ferroptosis (Du et al., 2020; Terzi et al., 2021). Maintaining a high level of NFS1 gene expression appears essential for survival and growth of cancer cells in high oxygen environment such as cells in a lung tumor or cancer cells during metastasis. Elevated oxygen concentration is more likely to damage ISC in cancer cells that have lower NFS1 expression, causing ferroptotic cell death (Alvarez et al., 2017). Paradoxically, in hypoxic tumor microenvironment, depletion of NFS1 was shown to display synthetic lethality with suppression of carbonic anhydrase IX (CAIX), likely due to that loss of CAIX activity decreased intracellular pH and increased ROS accumulation, which sensitized hypoxic tumor cells to ferroptosis (Chafe et al., 2021).

### Signaling pathways that connect mTORC1 activity with ferroptosis regulation

Cellular processes and signaling pathways that respond to changes in cell state or challenges from the extracellular environment play an important role in determining cellular metabolism. mTORC1 participates in many of these signaling events that ultimately result in metabolic perturbations associated with changes in ferroptosis susceptibility.

#### RTK oncogenic pathway

**Figure 2.**
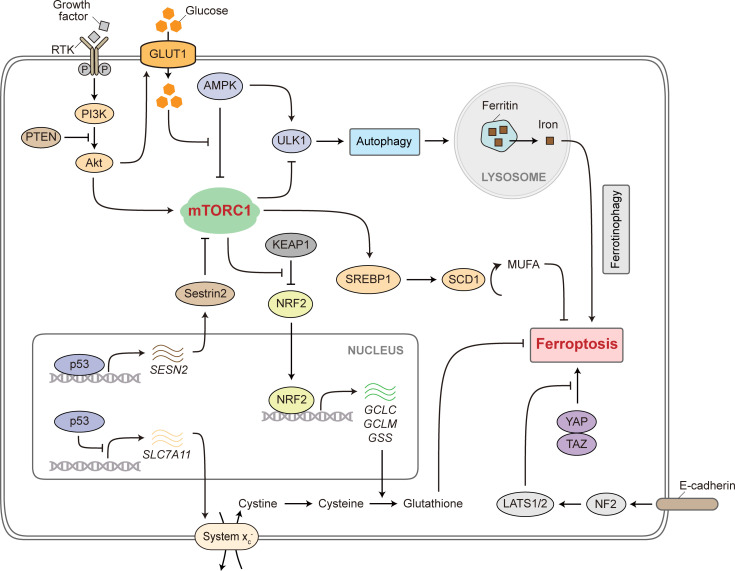
**Signaling pathways that connect mTORC1 activity with ferroptosis regulation.** mTORC1 is activated by growth factor signaling through the RTK-PI3K-Akt axis, of which one downstream outcome is the elevated MUFA production mediated by SREBP1 and SCD1. Growth factor signaling also promotes glucose uptake, which antagonizes AMPK activity. AMPK increases, while mTORC1 decreases, ULK1 activity that is required for autophagy initiation. Autophagic degradation of ferritin, a process termed ferrotinophagy, promotes ferroptosis by releasing iron from the intracellular iron store. The p53 tumor suppressor promotes expression of Sestrin2 that inhibits mTORC1 activity. On the other hand, p53 represses the expression of SLC7A11 required by the system x_c_^−^ plasma membrane antiporter, thereby promoting ferroptosis. Both these anti-mTORC1 and pro-ferroptosis roles are key components of the p53-mediated tumor suppressive function. mTORC1 promotes NRF2 activation by increasing KEAP1 degradation. NRF2 acts as a transcription factor that promotes the expression of enzymes involved in glutathione biosynthesis for ferroptosis suppression. Besides mTORC1, the Hippo pathway is another mechanism of cell size and growth regulation. The Hippo pathway effector proteins YAP and TAZ promote ferroptosis partly by increasing the expression of transferrin receptor and the acyl-CoA synthetase long chain family member 4 (ACSL4). Intercellular contact mediated by cadherin proteins led to NF2 activation and subsequently nuclear exclusion of YAP, thereby inhibiting ferroptosis. RTK, receptor tyrosine kinase. PI3K, phosphoinositide 3-kinase. PTEN, phosphatase and tensin homolog. GLUT1, glucose transporter 1 (encoded by *SLC2A1*). AMPK, AMP-activated protein kinase. ULK1, unc-51 like autophagy activating kinase 1. KEAP1, kelch-like ECH-associated protein 1. NRF2, nuclear factor E2-related factor 2. SREBP1, sterol regulatory element binding protein 1. SCD1, stearoyl-CoA desaturase 1. MUFA, monounsaturated fatty acid. NF2, also known as MERLIN. LATS1/2, large tumor suppressor kinase 1/2. YAP, Yes1 associated transcriptional regulator. TAZ, also known as WWTR1, WW domain containing transcription regulator 1.

Most cells in mammals acquire nutrients from the extracellular environment only when instructed by appropriate signaling cues. Activation of the RTK-PI3K-Akt axis in response to growth factor signaling allows uptake of nutrients such as glucose and amino acids (Fig. 2). In concert with increased nutrient acquisition, activation of mTORC1 downstream of the RTK-PI3K-Akt pathway promotes utilization of these nutrients for anabolic processes. This non-cell autonomous control of nutrient uptake forms the basis of coordinated nutrient distribution in multicellular organisms, and functions as a major barrier against tumorigenesis (Thompson, 2011).

Cancer cells are characterized with uncontrolled proliferation that depends on continuous nutrient supply. As a result, activating mutations in the RTK-PI3K-Akt pathway often occur in cancer to allow cell autonomous uptake of nutrients to support biomass accumulation and cell growth. A context-dependent role of RTK signaling in ferroptosis regulation has been suggested by recent studies. In non-small cell lung cancer, oncogenic alterations in the epidermal growth factor receptor (EGFR) increase cellular sensitivity to ferroptosis, due to mitogen-activated protein kinase (MAPK) signaling-mediated NADPH oxidase 4 (NOX4) activation (Poursaitidis et al., 2017). Similarly, in melanoma, activation of RTK signaling can result in resistance to MAPK-targeting therapies, but also leads to increased sensitivity to ferroptosis (Tsoi et al., 2018). By contrast, hepatocellular carcinoma (HCC) cells treated with sorafenib, a multi-target kinase inhibitor, were found to activate PI3K-RAC1-PAK1 signaling and increase macropinocytosis activity, and increased macropinocytosis prevented sorafenib-induced ferroptosis by replenishing the intracellular cysteine pool (Byun et al., 2022). It was also reported that sorafenib-resistant HCC cells upregulated the expression of fibronectin type III domain containing 5 (FNDC5), which in turn activated the PI3K-Akt pathway and promoted translocation of nuclear factor E2-related factor 2 (NRF2) into the nucleus to induce antioxidant response and ferroptosis resistance (Liu et al., 2022a). Inhibition of the RTK-PI3K axis potentiates immune checkpoint blockade therapy by sensitizing cancer cells to ferroptosis (Fan et al., 2021; Jiang et al., 2021b).

These contradicting observations regarding the role of RTK on ferroptosis regulation are likely a result of cell type- and context-specific response to RTK activation, as well as preferential usage of distinct signaling branches downstream of RTK. However, consensus appears to be achieved when concerning mTORC1 activation downstream of RTK pathway and its regulation on ferroptosis. As discussed, cancer cells carrying oncogenic mutations of PI3K or PTEN deletions were found to upregulate MUFA biosynthesis in an mTORC1-dependent manner, resulting in ferroptosis resistance (Yi et al., 2020; Fig. 2). Similarly, overexpression of GALNT14 in ovarian cancer confers resistance to chemotherapy induced ferroptosis by promoting EGFR and mTORC1 activity (Li et al., 2022). In agreement, other studies also indicated that mTORC1 activation led to ferroptosis resistance in multiple contexts of cancer, albeit likely involving mechanisms besides MUFA production, such as increased expression of the GPX4 protein (Ni et al., 2021; Zhang et al., 2021).

#### p53 tumor suppressive pathway

Loss of function mutations in *TP53* (encoding the p53 tumor suppressor) is frequently observed in human cancer. The tumor suppressive function of p53 is conventionally attributed to its role in promoting cell-cycle arrest, differentiation, apoptosis, or cellular senescence. These functions are achieved mainly through the transcriptional activity of p53 orchestrating the expression of a number of downstream target genes including *CDKN1A* (encoding p21) and *BBC3* (encoding PUMA). Over the past two decades, it has been demonstrated that regulation of cellular metabolism is another critical component of the p53 tumor suppressive function (Kruiswijk et al., 2015). For example, TP53-induced glycolysis and apoptosis regulator (TIGAR) harbors fructose-2,6-bisphosphatase activity and therefore reduces glycolytic activity when upregulated upon p53 stabilization (Bensaad et al., 2006). These findings are in line with the observation that p53 activation favors mitochondrial respiration as opposed to glycolysis for cellular bioenergetics, thereby counteracting Warburg effect seen in many tumors (Matoba et al., 2006). In addition to its role in modulating central carbon metabolism, p53 antagonizes mTORC1 activity by inducing the expression of sestrin proteins (Budanov and Karin, 2008; Fig. 2), thereby suppressing mTORC1-mediated anabolic metabolism under stress conditions such as DNA damage and nutrient deficit.

By mutating three lysine residues in the p53 DNA-binding domain into arginine (p53^3KR^, K177, K161, and K162 in mice) that abolishes post-translational acetylation of p53 at these sites, p53 lost the ability to induce cell-cycle arrest, apoptosis, or senescence (Li et al., 2012). However, p53^3KR^ still retained its tumor suppressive function as assessed by the absence of early-onset spontaneous tumors observed in p53^3KR^ knock-in mice, whereas their p53 null counterparts rapidly developed tumors within 6 mo after birth (Li et al., 2012). It turns out that p53 activation can suppress *SLC7A11* expression and therefore facilitate ferroptosis induction (Fig. 2). The p53^3KR^ mutant retains this ferroptosis-promoting activity, which likely accounts for part of its tumor suppressive function (Jiang et al., 2015). p53-mediated ferroptosis regulation was also observed in the context of immune cells and embryonic stem cells (Babaei-Abraki et al., 2022; Zhu et al., 2021). Mechanistically, in addition to direct p53 binding at the *SLC7A11* gene and suppressing its transcription, p53 promotes ferroptosis by inducing spermidine/spermine *N*^*1*^-acetyltransferase 1 (SAT1) expression that facilitates polyamine catabolism and peroxide generation (Ou et al., 2016). Furthermore, p53-mediated ferroptotic cell death seems to involve distinct lipid peroxidation modulation: while ACSL4-mediated PUFA-PL production leads to propagation of lipid ROS that can be cleared by GPX4, ACSL4 appears dispensable for p53-regulated ferroptosis. Instead, arachidonate 12-lipoxygenase (ALOX12) is required for lipid ROS generation during p53-dependent ferroptosis and tumor suppression (Chu et al., 2019). On the other hand, the calcium-independent phospholipase iPLA2β (encoded by *PLA2G6*) releases oxidized acyl tails from PL to suppress p53-driven ferroptosis (Chen et al., 2021b).

The role of p53 signaling in ferroptosis seems to be context-dependent and sometimes even opposite. For example, in colorectal cancer, p53 antagonizes erastin-induced ferroptosis by limiting dipeptidyl-peptidase-4 (DPP4) activity and reducing DPP4-dependent lipid peroxidation (Xie et al., 2017); activation of p21 downstream of p53 delays the onset of ferroptosis, partly due to conservation of intracellular GSH upon cell cycle arrest (Tarangelo et al., 2018; Venkatesh et al., 2020). Although these studies seem contradictory regarding the role of p53 in ferroptosis regulation, the decision on cell survival versus cell death has long been debated in p53 biology (Kruiswijk et al., 2015). The outcome of p53 activation is a result of multifactorial intracellular and extracellular inputs, and usually reflects an optimal cellular response to specific genetic and environmental alterations.

Intriguingly, lysine 98 was identified as an additional residue on top of p53^3KR^ whose acetylation is critical for p53-mediated ferroptosis induction (Wang et al., 2016). Furthermore, p53 defective in acetylation at all these four lysine residues (p53^4KR^, K98, K177, K161, and K162 in mice) lost the ability to induce cell cycle arrest, apoptosis, senescence, or ferroptosis, but still partially protected mice from developing spontaneous early onset tumors. The residual tumor suppressive function was suggested to be due to the ability of p53^4KR^ to inhibit mTORC1 through upregulating sestrin proteins (Fig. 2), which prevented excessive cell proliferation (Kon et al., 2021). Together, these findings suggest that metabolic checkpoints, such as mTORC1 activity and ferroptotic cell death regulation, are fundamental determinants of p53-mediated tumor suppression.

#### AMPK signaling pathway

AMP-activated protein kinase (AMPK) acts as a major sensor of cellular energy status through its direct interaction with ATP, ADP, or AMP. A decrease in cellular energy often leads to increased AMP:ATP ratio, which potently activates AMPK to phosphorylate its downstream protein targets. AMPK activation favors cellular catabolic processes in order to cope with nutrient and energy deficit (Trefts and Shaw, 2021). For example, AMPK phosphorylates a number of regulatory proteins, including ULK1, BECLIN-1, and ATG9, to initiate macroautophagy (referred to as autophagy hereafter) and maintain cellular homeostasis under nutrient starvation (Egan et al., 2011; Kim et al., 2013; Kim et al., 2011; Weerasekara et al., 2014; Fig. 2). By contrast, AMPK can also phosphorylate acetyl-CoA carboxylase (ACC) and inhibit its activity required for de novo fatty acid biosynthesis (Fullerton et al., 2013; Munday et al., 1988). Furthermore, AMPK activation suppresses mTORC1 activity through phosphorylation of TSC2 that possibly promotes its GAP activity toward Rheb (Inoki et al., 2003), as well as through inhibitory phosphorylation of the RAPTOR scaffold protein which leads to reduced S6K1 and 4EBP phosphorylation downstream of mTORC1 (Gwinn et al., 2008).

By inhibiting mTORC1 activity, AMPK dampens nutrient- and energy-demanding anabolic processes such as protein translation, lipid biosynthesis, and nucleotide production. Regulation of mTORC1 activity by AMPK appears to be a fundamental mechanism that signals nutrient insufficiency to the inhibition of cell growth, and is conserved even in plants and budding yeast (Hughes Hallett et al., 2015; Nukarinen et al., 2016; Wu et al., 2019b). Conversely, mTORC1 directly phosphorylates the AMPK catalytic subunit and downregulates its activity independent of AMP:ATP ratio change (Ling et al., 2020). The antagonistic crosstalk between AMPK and mTORC1 is essential in maintaining cellular metabolic homeostasis (Gonzalez et al., 2020). Notably, lysosomes have emerged as an important subcellular location where precise regulation of both AMPK and mTORC1 activity occurs (Lawrence and Zoncu, 2019; Lin and Hardie, 2018). In particular, AXIN acts as a scaffold protein on the lysosomal surface that tethers LKB1 (encoded by *STK11*) to phosphorylate and activate AMPK (Zhang et al., 2013). By forming a complex with v-ATPase and Ragulator upon energy stress, AXIN-LKB1-AMPK inactivates mTORC1 and displaces it from the lysosome (Zhang et al., 2014).

While the function of AMPK in antagonizing mTORC1 and promoting catabolism is well recognized, its role in ferroptosis regulation appears to be controversial. On one hand, activation of AMPK promotes ferroptosis. This might be achieved through its phosphorylation of BECLIN-1 to interfere with system x_c_^−^ activity, leading to reduced cystine uptake (Song et al., 2018). Additionally, AMPK facilitates ferroptosis by downregulating SREBP1- and SCD1-dependent MUFA production (Zhao et al., 2020b). These findings are consistent with observations in multiple lung adenocarcinoma models bearing *STK11* and *KEAP1* mutations, where elevated expression of SCD1, due to inactivated LKB1-AMPK signaling, resulted in ferroptosis resistance (Wohlhieter et al., 2020). On the other hand, by suppressing PUFA biosynthesis through inactivating ACC, AMPK inhibits ferroptosis (Lee et al., 2020; Li et al., 2020). Therefore, the context-specific outcome might partly result from a differential contribution of MUFA and PUFA production to ferroptosis regulation in response to AMPK signaling. Further mechanistic details, including whether mTORC1 is directly involved in ferroptosis regulation by AMPK, are yet to be determined.

#### KEAP1-NRF2 pathway

As described, mTORC1 can engage multiple oncogenic and tumor suppressive pathways, as well as anabolic and catabolic processes to regulate ferroptosis. Nuclear factor erythroid 2-related factor 2 (NRF2) is a transcription factor that promotes the expression of a cohort of antioxidant response genes (Fig. 2). NRF2 protein level is controlled by the kelch-like ECH-associated protein 1 (KEAP1) E3 ubiquitin ligase. Loss of function mutations of KEAP1 are recurrently found in various types of cancer (Martincorena et al., 2017). mTORC1 directly phosphorylates the autophagic cargo adaptor protein p62, which mediates KEAP1 degradation and therefore NRF2 activation (Ichimura et al., 2013; Woo et al., 2019). As a central coordinator of cellular antioxidant response, NRF2 activity has been associated with ferroptosis resistance primarily due to its role in upregulating genes involved in GSH biosynthesis (Rojo de la Vega et al., 2018; Fig. 2), although non-conventional functions of these canonical NRF2 targets have also been suggested to prevent ferroptosis independent of GSH production (Kang et al., 2021).

More recently, additional downstream gene targets of NRF2 have emerged as important regulators of ferroptosis. Aldo-keto-reductase 1C (AKR1C) family members were identified as NRF2-regulated ferroptosis-protective genes, and their increased expression underlies the progression of lung cancers with concurrent *KEAP1* and *STK11* mutations (Wohlhieter et al., 2020). Similarly, NRF2 transcriptionally activates ferroptosis suppressor protein 1 (FSP1), which mediates ferroptosis- and radiation-resistance in *KEAP1*-mutated lung cancer cells (Koppula et al., 2022). In pancreatic cancer cells, microsomal glutathione S-transferase 1 (MGST1) was upregulated in response to ferroptosis induction in an NRF2-dependent manner, which in turn, functions to suppress ferroptosis by limiting lipid peroxidation (Kuang et al., 2021). The amino acid tryptophan was often depleted in the tumor microenvironment due to its catabolism by tryptophan 2,3-dioxygenase (TDO2) or indoleamine 2,3-dioxygenase (IDO1), resulting in the production of kynurenine. Kynurenine produced by infiltrating myeloid cells was found to be taken up by surrounding cancer cells in the tumor microenvironment and contributed to ferroptosis resistance in an NRF2-dependent fashion (Fiore et al., 2022).

#### Hippo pathway

In addition to mTORC1 signaling, the Hippo pathway is another mechanism regulating cell size and growth. As a result, multiple studies have revealed an extensive interplay between mTORC1 and the Hippo pathway in coordinating cellular metabolism (Koo and Guan, 2018). Intercellular contact mediated by cadherin proteins led to NF2 (also known as MERLIN) activation and subsequently nuclear exclusion of the YAP transcription coactivator in the Hippo pathway. Transferrin receptor 1 (TFRC) and ACSL4 were both identified as transcriptional targets of YAP that mediate ferroptosis. Consequently, increased cell–cell interaction was found to promote ferroptosis resistance as YAP-mediated transcription was reduced (Wu et al., 2019a; Fig. 2). The results were further confirmed in the context of renal cell carcinoma that activation of the Hippo pathway effector TAZ also promoted ferroptosis (Yang et al., 2019b). These findings were reminiscent of the observations made in studies of therapy-resistant cancer cells that drug-tolerant persister cells often adopt a high-mesenchymal state and are particularly vulnerable to ferroptotic cell death (Hangauer et al., 2017; Viswanathan et al., 2017).

#### Epigenetic regulation

Versatile functions of mTORC1 also include the regulation of cellular epigenetic process, an important determinant of cell fate such as cell growth, differentiation, and death (Chi, 2012; Pfister and Ashworth, 2017). Epigenetic enzymes such as histone acetyltransferases and methyltransferases use acetyl-CoA and SAM as acetyl and methyl donors, respectively, to modify histones as well as nucleic acids, leading to changes of chromatin structure and gene expression. Therefore, mTORC1 can impinge on epigenetic machinery either through its role in cellular metabolism to alter acetyl-CoA and SAM availability or through its protein kinase activity to directly phosphorylate key epigenetic enzymes. For instance, histone acetyltransferase p300 is a substrate of mTORC1, and mTORC1-mediated phosphorylation of p300 has been shown to activate the transcription of multiple lipogenesis genes (Wan et al., 2017); mTORC1 activation was reported in several human glioblastoma cell lines to upregulate EZH2, the main component of H3K27-specific polycomb repressive complex 2 (PRC2) methyltransferase (Harachi et al., 2020); and in neural stem cell development, mTORC1 inhibition upregulated the expression of DNA methyltransferases DNMT1 and DNMT3, hence increasing DNA methylation of the neuron-specific gene neurogenin 1 (*NEUROG1*; Zhang et al., 2017). Conversely, epigenetic enzymes can modulate mTORC1 activity by altering cellular metabolism or expression of proteins involved in mTORC1 regulation. For example, H3K27me3 deposited by EZH2 suppresses the transcription of genes encoding negative regulators of mTORC1 such as *TSC2* and *DEPTOR*, rendering enhanced mTORC1 activity to attenuate autophagy (Wei et al., 2015); and intriguingly, the fat mass and obesity-associated protein (FTO) catalyzes m^6^A demethylation in a Fe (II)- and α-KG-dependent manner (Jia et al., 2011), and depletion of FTO in mouse embryonic fibroblasts can weaken the activation of the mTORC1 pathway, leading to enhanced autophagy (Gulati et al., 2013).

Although these studies did not directly link epigenetics with ferroptosis regulation, the cellular processes impacted by epigenetic pathways, such autophagy and lipogenesis, as well as a variety of metabolites involved in epigenetic regulation, such as acetyl-CoA, α-KG and the transit metal Fe (II), are all established regulators of ferroptosis. These mutual regulatory mechanisms between mTORC1 activity and epigenetic pathways suggest a potential functional interaction between epigenetic process and ferroptosis that warrants further investigations.

#### Autophagy

Autophagy is a catabolic process that recycles intracellular macromolecules and defective organelles through lysosomal degradation in response to a variety of stress. In contrast to a positive regulatory role of AMPK on autophagy as discussed above, mTORC1 is known to restrict autophagy through multiple mechanisms (Fig. 2). mTORC1 phosphorylates ULK1 and ATG13, and inhibits the correct formation of the autophagosome initiated by the ULK1-ATG13-FIP200 complex (Ganley et al., 2009; Hosokawa et al., 2009). mTORC1 also phosphorylates ULK1 at Serine 757 to disrupt ULK1 interaction with AMPK (Hosokawa et al., 2009; Kim et al., 2011). In addition to the inhibitory effect on autophagosome assembly, mTORC1 phosphorylates the transcription factor EB (TFEB), a critical regulator of lysosome biogenesis, to prevent its nuclear translocation required for lysosomal biogenetic gene transcription (Martina and Puertollano, 2013; Roczniak-Ferguson et al., 2012).

Through quantitative proteomics analysis, the nuclear receptor coactivator 4 (NCOA4) was identified as a cargo receptor essential for the autophagic degradation of ferritin (Mancias et al., 2014). This process, termed ferritinophagy, was found to release iron from ferritin to promote ferroptosis (Gao et al., 2016; Hou et al., 2016; Fig. 2). These findings were consistent with the observation in pancreatic cancer cells, that inhibition of GOT1 promoted cellular catabolism and increased intracellular labile iron content to facilitate ferroptosis (Kremer et al., 2021). Furthermore, autophagic degradation of the circadian rhythm regulator, BMAL1 (encoded by *ARNTL*), promoted ferroptosis through EGLN2-mediated HIF1α degradation (Yang et al., 2019a). Chaperone-mediated autophagy was reported to contribute to ferroptosis by targeting GPX4 for degradation (Chen et al., 2021a). It should be noted that there are also contexts in which autophagy might be protective against ferroptosis. For example, in human airway epithelial cells, concomitant activation of autophagic pathways, in particular PE-binding protein 1 (PEBP1)-mediated LC3 lipidation, was observed upon pro-ferroptotic cell death signals to protect cells from ferroptosis (Zhao et al., 2020a).

### mTORC1 in other forms of cell death

Besides ferroptosis, cellular metabolism also plays a deterministic role during other forms of regulated cell death. Apoptosis has long been recognized as a programmed cell death process involving extensive interaction with cellular metabolism. More recently, pyroptosis has emerged as a distinct cell death mode also heavily regulated by metabolic pathways. In this section, we review the role mTORC1 plays in regulating apoptosis and pyroptosis.

#### mTORC1 in apoptosis regulation

**Figure 3.**
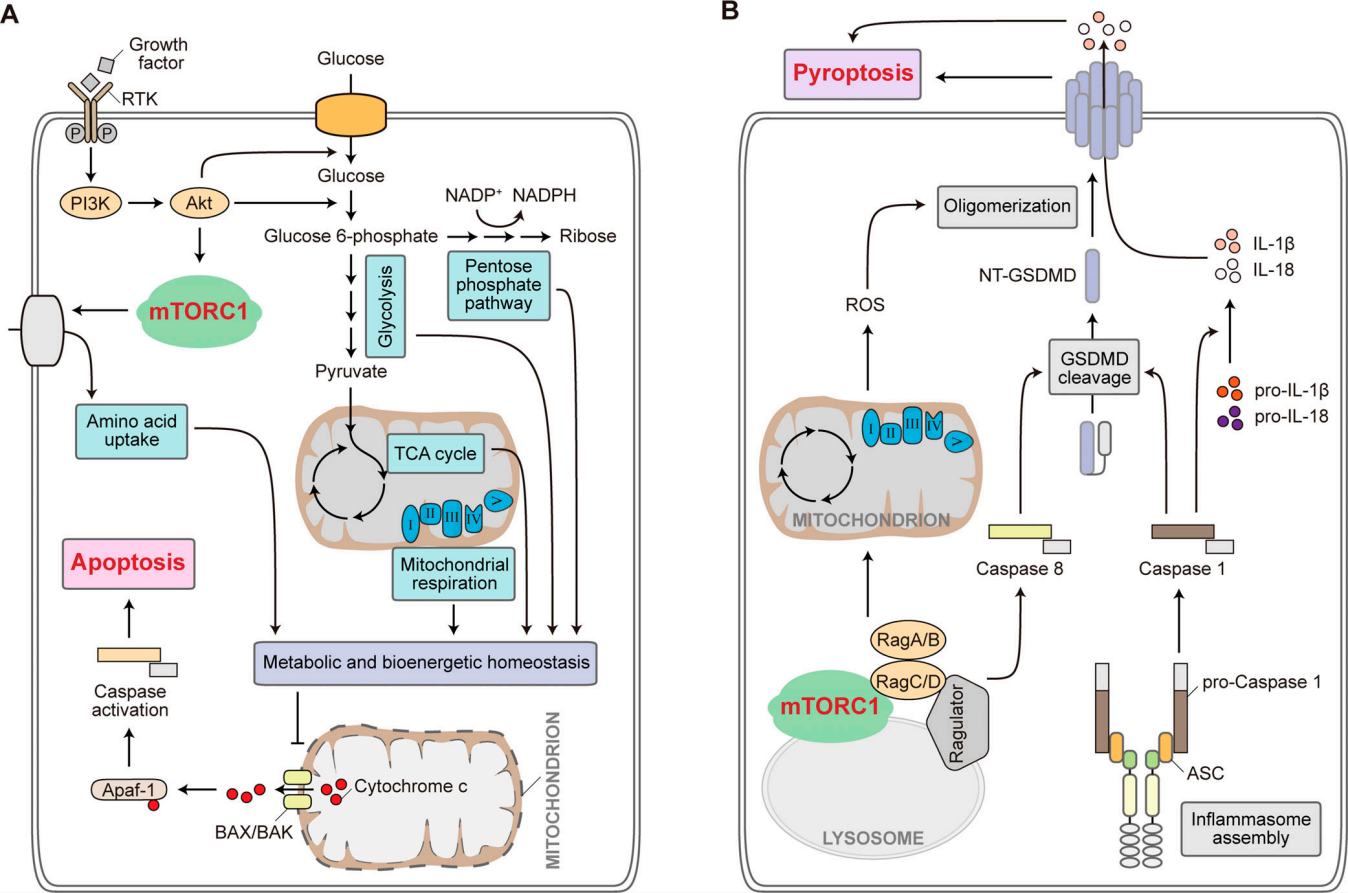
**mTORC1 in apoptosis and pyroptosis. (A)** mTORC1 in apoptosis regulation. Intracellular metabolic and bioenergetic homeostasis is maintained through coordinated actions of multiple metabolic processes, including nutrient uptake, glycolysis, pentose phosphate pathway, TCA cycle, and mitochondrial respiration. mTORC1 participates in the regulation of a variety of these metabolic pathways. Disruption of metabolic homeostasis can promote the intrinsic mitochondrial apoptotic pathway, where activation of BAX and BAK facilitates the release of cytochrome c from mitochondria into the cytosol. Cytochrome c then forms apoptosome with Apaf-1, which in turn activates downstream caspases to promote apoptosis. RTK, receptor tyrosine kinase. PI3K, phosphoinositide 3-kinase. Apaf-1, apoptotic peptidase activating factor 1. BAX, BCL2 associated X, apoptosis regulator. BAK, also known as BAK1, BCL2 antagonist/killer 1. **(B)** mTORC1 in pyroptosis regulation. Inflammasome assembly stimulated by a variety of pathogen-associated molecular patterns (PAMPs) recruits caspase 1, which in turn proteolytically activates IL-1β and IL-18. Activated caspase 1 also leads to GSDMD cleavage required for pyroptosis. Components of the Ragulator-Rag-mTORC1 pathway are required for GSDMD oligomerization and plasma membrane pore formation during pyroptosis, in a manner dependent on the production of mitochondrial ROS. Independent of mTORC1, Ragulator-Rag also promotes caspase 8-dependent GSDMD cleavage to facilitate pyroptosis in the presence of the pathogenic bacteria *Yersinia*. ROS, reactive oxygen species. GSDMD, gasdermin D. NT-GSDMD, N-terminus of gasdermin D. ASC, also known as PYCARD, PYD and CARD domain containing.

Apoptosis, particularly the mitochondrial apoptotic pathway, involves extensive crosstalk with the cellular metabolic network (Fig. 3 A). Mitochondrion hosts a variety of metabolic reactions and is also the organelle that initiates cell intrinsic apoptosis through BAX and BAK activation followed by mitochondrial outer membrane permeabilization and cytochrome c release (Bock and Tait, 2020; Green, 2022a; Green, 2022b; Fig. 3 A). Metabolic regulation of apoptosis is further evidenced by the observation that growth factor withdrawal led to apoptosis through downregulated nutrient uptake (Rathmell et al., 2000), and that cell-autonomous nutrient import upon oncogene activation (such as constitutive Akt activation) was sufficient to prevent BAX-mediated apoptosis (Rathmell et al., 2003). Further studies revealed that glycogen synthase kinase-3 (GSK-3) activity was suppressed upon Akt activation, which resulted in reduced phosphorylation of Mcl-1, a member of the antiapoptotic Bcl-2 family proteins. Reduced Mcl-1 phosphorylation prevented it from ubiquitination-mediated degradation and therefore cells were protected against apoptosis (Ding et al., 2007; Maurer et al., 2006). mTORC1 might also play a role in this process. Reduced glucose utilization in the absence of growth factor signaling results in AMPK activation and mTORC1 inhibition, which sensitized cells to apoptosis by downregulating protein translation of Mcl-1 (Pradelli et al., 2010). Consistently, caloric restriction or rapamycin treatment, both known to suppress mTORC1 activity, sensitize lymphoma and leukemia cells to apoptosis through reduced expression of Mcl-1 (Meynet et al., 2013; Wei et al., 2006).

By contrast, multiple studies have proposed a role of mTORC1 in promoting apoptosis, which is likely achieved through the action of mTORC1 signaling in balancing apoptosis with autophagy. Constitutive mTORC1 activation, such as upon TSC loss or in the context of melanoma cells carrying oncogenic activation of Ras signaling, was shown to display increased vulnerability to apoptosis induction as a result of mTORC1-mediated suppression of autophagy (Gremke et al., 2020; Ng et al., 2011; Sheen et al., 2011). In addition, mTORC1 stimulation by excessive glutaminolysis was found to confer apoptosis sensitivity in cancer cells, and that rapamycin treatment rescued cell viability by stimulating autophagy (Villar et al., 2017). In pancreatic β-cells, exposure to high levels of glucose and lipids, as well as activation of LATS2 in the Hippo signaling pathway, stimulates mTORC1 and inhibits autophagy, thus contributing to β-cell apoptosis and the development of diabetes (Mir et al., 2015; Yuan et al., 2021). Similarly, protein-rich diets elevate amino acid levels in blood, which activate mTORC1 and suppress mitochondrial autophagy in macrophages, resulting in macrophage apoptosis and atherosclerotic progression (Zhang et al., 2020). It appears that mild stress conditions often suppress mTORC1 to induce autophagy, eliciting a protective cellular response; however, as stress proceeds and exceeds a lethal threshold, this protective mechanism is overwhelmed and a cell death program, be it apoptotic or other death modalities, is activated (Marino et al., 2014).

#### mTORC1 in pyroptosis regulation

In distinct contrast to ferroptosis and apoptosis, pyroptosis is a pro-inflammatory form of regulated cell death that typically takes place in cells of myeloid lineage (Bergsbaken et al., 2009; Jorgensen and Miao, 2015). The execution of pyroptosis is often proceeded by inflammasome assembly. Several inflammasome-forming receptors have been identified, including NLR family pyrin domain containing 1 and 3 (NLRP1 and NLRP3), NLR family CARD domain containing 4 (NLRC4), absent in melanoma 2 (AIM2) and pyrin (encoded by *MEFV*). These pattern-recognition receptor proteins can initiate the assembly of distinct inflammasomes in response to a variety of pathogen-associated molecular patterns (PAMPs). The assembled complex recruits and activates downstream caspases (often caspase-1) to proteolytically process-specific interleukins (IL-1β and IL-18) for their maturation and release, and to proteolytically activate pore-forming gasdermin proteins, resulting in ultimate inflammatory response and pyroptotic cell death (Broz and Dixit, 2016; Fig. 3 B).

mTORC1 signaling has been implicated in the regulation of inflammasome activation in multiple contexts. For example, interleukin-10 (IL-10) suppresses mTORC1 activity through STAT3-mediated DDIT4 upregulation, which, in turn, promotes mitophagy in macrophages. This accelerated mitochondrial turnover prevented activation of the NLRP3 inflammasome and reduced IL-1β production, resulting in a net anti-inflammatory effect exerted by these macrophages (Ip et al., 2017). Similarly, elevated expression of the AIM2 inflammasome was seen in T_reg_ cells upon transforming growth factor β (TGFβ) induction, which attenuated Akt-mTORC1 signaling and restrained autoimmune response, although this immune-repressive effect of AIM2 observed in T_reg_ cells appeared to be independent of its typical pro-inflammatory role in myeloid cells (Chou et al., 2021). These and other findings in general suggest that mTORC1 functions to promote inflammasome activation (de la Roche et al., 2018; Marin-Aguilar et al., 2020; Nazir et al., 2017), whereas an inhibitory role of mTORC1 has also been reported during the activation of pyrin inflammasome (Sharma et al., 2020).

Consistent with its regulatory role for inflammasome activation, the mTORC1 signaling pathway directly participates in pyroptosis regulation. A forward genetic screen performed in macrophages revealed that components of the Ragulator-Rag-mTORC1 pathway were essential for gasdermin D (GSDMD) oligomerization and the subsequent plasma membrane pore formation during pyroptosis (Evavold et al., 2021). Further studies indicated that the pyroptosis-promoting effect of mTORC1 was independent of GSDMD cleavage, but was largely mediated by the production of mitochondrial ROS (Evavold et al., 2021; Fig. 3 B). On the other hand, macrophage infection by the pathogenic bacteria, *Yersinia*, induces receptor-interacting serine-threonine protein kinase 1 (RIPK1)- and caspase 8-mediated GSDMD cleavage and pyroptosis (Orning et al., 2018; Sarhan et al., 2018). The Ragulator-Rag complex is required during this caspase-8-dependent, but inflammasome-independent form of pyroptosis (Zheng et al., 2021; Fig. 3 B). In response to *Yersinia*, Ragulator-Rag functions as a tethering platform at the lysosome for recruitment and activation of the RIPK1-caspase-8 complex. The Rag GTPase activity, but not mTORC1 activity, was found essential in promoting RIPK1-caspase-8-induced pyroptosis (Zheng et al., 2021). Although mechanistic details of how distinct components in the Ragulator-Rag-mTORC1 signaling cascade modulate pyroptotic cell death are yet to be fully understood and are likely dependent on the specific contexts, these findings are consistent with each other in suggesting that the Ragulator-Rag complex acts as a control node in determining whether adequate nutrients are available to support cell proliferation versus if a cell should commit pro-inflammatory cell death in case of pathogen infection. In agreement with an overall pyroptosis-promoting role of the mTORC1 signaling pathway, inhibiting mTORC1 by Sestrin2 expression or by rapamycin was reported to suppress NLRP3 inflammasome activation and ameliorate pyroptosis-associated diseases such as cholestatic liver injury and sleep deprivation induced renal damage (Han et al., 2022; Liu et al., 2022b).

### Therapeutic opportunities by targeting mTORC1 and its role in ferroptosis regulation

With an increasing knowledge in understanding ferroptosis, the potentials of modulating ferroptosis in treating various human diseases have been extensively investigated. Studies of these disease implications have been largely facilitated by the development and optimization of ferroptosis inducers and inhibitors that target different regulatory steps of ferroptosis. Ferroptosis was first defined through a screen of small molecules targeting cancer cells, from which erastin was identified to induce lipid oxidation and ultimate ferroptosis by inhibiting system x_c_^−^ (Dixon et al., 2012). Imidazole ketone erastin (IKE) was developed as a more potent and stable derivative of erastin that displays antitumor effect in mouse models (Zhang et al., 2019). Sulfasalazine, a well-documented drug used in the clinic for the treatment of autoimmune diseases, was also reported to exert a modest inhibitory activity on system x_c_^−^ (Gout et al., 2001; Ishimoto et al., 2011). Furthermore, the kinase inhibitor sorafenib, approved for the treatment of liver, thyroid, and kidney tumors, was demonstrated to function as a cytotoxic agent partly through system x_c_^−^ inhibition and ferroptosis induction (Lachaier et al., 2014; Louandre et al., 2013). In addition to targeting system x_c_^−^, small molecules that perturb the function of GPX4 are also potent inducers of ferroptosis. RSL3 is one of the first described such chemicals that inactivate GPX4 by covalent interaction with its catalytic site (Yang et al., 2016; Yang et al., 2014). Moreover, FIN56 was also demonstrated as a ferroptosis inducer that acts by promoting GPX4 degradation (Shimada et al., 2016).

A number of ferroptosis-inhibitory chemicals have also been identified. Ferrostatin-1 (Fer-1) was reported to antagonize erastin-induced ferroptosis (Dixon et al., 2012). Similarly, a spiroquinoxalinamine derivative, liproxstatin-1 (Lip-1), was identified as an anti-ferroptosis molecule with better pharmacokinetic profiles than Fer-1 (Friedmann Angeli et al., 2014). Both Fer-1 and Lip-1 act as lipid ROS scavengers that are reminiscent of the function of natural antioxidants such as vitamin E. However, Fer-1 and Lip-1 were demonstrated to be more potent and selective against ferroptosis than vitamin E derivatives (Zilka et al., 2017). In addition to these radical trapping agents (RTAs), other ferroptosis-inhibitory approaches that target ACSL4, lipoxygenases, or intracellular labile iron are being actively pursued (Seibt et al., 2019). These molecules with anti-ferroptotic properties hold promise in ameliorating pathological conditions for which blocking ferroptosis-mediated loss of cells and tissues is desired.

While ferroptosis modulating agents are only starting to be characterized in recent years, investigations on mTOR inhibitors date back to the 1970s when rapamycin was isolated (Vezina et al., 1975). Studies in the 1990s have led to the approved clinical use of rapamycin in preventing allograft rejection during renal transplantation, as well as in anti-restenosis during coronary angioplasty stents (Andoh et al., 1996; Thompson, 2003). Since the discovery of mTOR as the target of rapamycin in the 1990s, as well as the characterization of mTOR complexes in multiple biological processes in the following decades, the use of rapamycin and its analogues (rapalogs) has been widely studied in various disease contexts including cancer, neurodegeneration, metabolic syndromes, and age-related disorders. In addition to rapalogs acting as allosteric inhibitors of mTORC1, ATP analogues that compete with ATP binding at the mTOR kinase domain were also developed. These ATP-competitive catalytic inhibitors target both mTORC1 and mTORC2, and often display a more sustained mTOR inhibitory effect compared with that of rapalogs reported in certain preclinical settings. However, inhibition of mTORC2 can lead to side effects including insulin resistance (Kleinert et al., 2014; Lamming et al., 2012).

Cancer therapeutic potential of targeting mTORC1-ferroptosis connection has been extensively investigated. Ferroptosis is a potential intrinsic tumor suppressive mechanism, and induction of ferroptosis is a critical component of the tumor suppressive activity of p53 (Jiang et al., 2015). Other tumor suppressive pathways were also reported to associate with cellular susceptibility to ferroptosis induction. For example, loss of function mutations in the E-cadherin-NF2-Hippo pathway was frequently observed in various types of cancer including mesothelioma (Harvey et al., 2013). Mutations in the NF2 tumor suppressor gene lead to increased YAP activity which promotes ACSL4 and TFRC expression to facilitate ferroptosis progression. As a result, NF2-deficient mesothelioma is exquisitely sensitive to ferroptosis induction by genetic or pharmacologic approaches (Wu et al., 2019a).

Importantly, oncogenic signaling pathways converging on mTORC1 often result in enhanced resistance to ferroptosis. As detailed above, mTORC1 activation leads to ferroptosis resistance through mechanisms including elevated MUFA biosynthesis and increased GPX4 protein translation (Yi et al., 2020; Zhang et al., 2021). In mouse xenograft models of breast cancer or prostate cancer that harbors PI3K or PTEN mutation, respectively, combining the use of IKE and the rapalog CCI-779 led to substantial regression of established tumors (Yi et al., 2020). Similarly, lung cancer patient-derived xenograft (PDX) tumors were found highly sensitive to a combination of mTORC1 inhibition and ferroptosis induction by AZD-8055 (ATP-competitive mTOR inhibitor) and IKE, respectively (Zhang et al., 2021). In addition, mTORC1 inhibition by torin-2 synergizes with Fin56 in promoting bladder cancer cell ferroptosis (Sun et al., 2021). Based on results from these pre-clinical cancer models, it is conceivable that combinatorial therapies involving mTORC1 inhibition and ferroptosis induction can be developed as clinical treatment in a variety of cancer contexts.

In contrast to cancer treatment where elimination of malignant cells by ferroptosis is desired, other pathological conditions, such as neurodegeneration and ischemia-reperfusion injury, often result from aberrant loss of normal cells that is in part due to ferroptosis. Therefore, inhibition of ferroptosis might mitigate the pathologies associated with these diseases (see text box). It is yet to be determined whether functional interaction between mTORC1 and ferroptosis contributes to the progression of neurodegeneration and ischemia-reperfusion injury, and whether combinatorial perturbation of mTORC1 and ferroptosis activity might be therapeutically beneficial in treating these diseases.

Neurodegeneration and ischemia-reperfusion injury

**Neurodegeneration**

Neuron-specific deletion of GPX4 or ablation of selenocysteine tRNA (tRNA[Ser]^Sec^) was reported to cause neurodegeneration in mice (Seiler et al., 2008; Wirth et al., 2010). The observed neuronal toxicity accompanied with elevated lipid peroxidation was later recognized as ferroptotic cell death, providing early evidence that ferroptosis can contribute to neurodegenerative diseases (Seiler et al., 2008). Subsequent studies suggest that ferroptosis plays a role in promoting the development of Alzheimer’s disease, Huntington’s disease, and Parkinson’s disease likely resulting from dysregulated iron metabolism and increased oxidative stress in these disease states (Do Van et al., 2016; Skouta et al., 2014; Zhang et al., 2018). In agreement, iron chelation as well as lipid ROS scavengers were found to mitigate these neurological pathologies (Devos et al., 2014; Skouta et al., 2014; Weinreb et al., 2013).

The connection between mTORC1 and neurodegeneration has long been established and is associated with the role of mTORC1 in aging. Reduced mTORC1 activity, through direct inhibition by rapalogs or indirectly through dietary restriction, has been reported as a unifying mechanism for healthy lifespan extension conserved in diverse species (Green et al., 2022). This overall improved health state and anti-aging effect of mTORC1 inhibition is thought to contribute to the prevention of neurodegenerative pathologies. Furthermore, neurodegenerative diseases are often characterized with the appearance of protein aggregates that lead to defective neuronal function, such as accumulation of amyloid-β (Aβ) in Alzheimer’s disease, α-synuclein-containing inclusion bodies (Lewy bodies) in Parkinson’s disease, as well as HTT with polyglutamine tract in Huntington’s disease. Inhibition of mTORC1 by rapalogs was found to activate autophagy and promote clearance of these toxic aggregates, and was generally shown to improve treatment of neurodegenerative diseases (Bove et al., 2011; Crino, 2016; Lipton and Sahin, 2014).

**Ischemia-reperfusion injury**

Reperfusion is essential for the protection of ischemic tissues from infarction. However, the process of ischemia-reperfusion is often associated with acute reoxygenation and elevated oxidative stress that can lead to irreversible tissue injury. Ferroptosis plays a major role in mediating cell death during ischemia-reperfusion injury in the kidney and in the heart (Fang et al., 2019; Friedmann Angeli et al., 2014; Gao et al., 2015; Linkermann et al., 2014). In accordance, lipid ROS scavengers, as well as ferroptosis inhibition by perturbing glutaminolysis, were shown to mitigate organ damage induced by ischemia-reperfusion (Friedmann Angeli et al., 2014; Gao et al., 2015; Linkermann et al., 2014).

During the reperfusion phase following ischemia, multiple studies have highlighted a protective role of mTORC1 by mechanisms such as suppressing apoptosis and maintaining endothelial integrity (Grahammer et al., 2014; Gui et al., 2019; Kong et al., 2016; Oka et al., 2017). However, there is also evidence that inhibiting mTORC1 may be beneficial in restraining ischemia-reperfusion injury under certain circumstances (Samidurai et al., 2020). The controversial results are reflective of the acute and dynamic features of the ischemia-reperfusion injury process, which are important to consider when designing therapeutic interventions that target mTORC1 as well as ferroptosis.

### Perspectives

Extensive interactions between mTORC1 signaling and cell death regulation mediated by a variety of intracellular metabolites and metabolic pathways have been revealed in the past decades, yet numerous mechanistic details remain to be characterized. Our knowledge in how mTORC1 participates in the regulation of various forms of cell death has led to promising strategies for the treatment of relevant pathologies. It is hopeful that many of these therapeutic paradigms may come to use in the next phase of our endeavor in this research field.
